# Stability-Indicating LC Method for the Determination of Prasugrel Hydrochloride in Pharmaceutical Dosage Form

**DOI:** 10.3797/scipharm.1201-05

**Published:** 2012-03-20

**Authors:** Vinod K. Ahirrao, Chabutai S. Patil, Saroj B. Bembalkar, Sanjay B. Ubale, Rajendra P. Marathe, Rajesh. B. Nawale, Mahadev G. Landge, Rajendra. P. Pawar

**Affiliations:** 1 Department of Chemistry, Deogiri College, Aurangabad-431004 (MS), India; 2 Government college of Pharmacy, Aurangabad-431004 (MS), India; 3 Department of Chemistry, Vidyanath College, Parli-vaijnath-431515, India

**Keywords:** Liquid chromatography, Method validation, Pharmaceutical preparation, Prasugrel hydrochloride

## Abstract

A simple, rapid and precise method was developed for the quantitative estimation of prasugrel hydrochloride in pharmaceutical dosage form. A chromatographic separation of prasugrel and its degradants was achieved with Zorbax XDB C_8_, 150 × 4.6 mm, 3.5μm analytical column using aqueous solution of 0.05 M ammonium acetate pH 4.5 with acetic acid-acetonitrile (40:60 *v/v*). The instrumental settings include flow rate of 1.0 ml/min, column temperature at 30°C and detector wavelength of 254 nm using a photodiode array detector. Theoretical plates for prasugrel were 7023. Tailing factor for prasugrel was 1.11. Prasugrel was exposed to thermal, photolytic, hydrolytic and oxidative stress conditions, and the stressed samples were analyzed by the proposed method. Peak homogeneity data of prasugrel was obtained using photodiode array detector in the stressed sample chromatograms, which demonstrated the specificity of the method for the estimation in presence of degradants. The described method showed excellent linearity over a range of 10–300 μg/ml for prasugrel. The correlation coefficient is 0.999. The relative standard deviation of peak area for six measurements is always less than 2%. Overall, the proposed method was found to be suitable and accurate for quantitative determination and stability study of prasugrel in pharmaceutical dosage form.

## Introduction

**Fig. 1.**
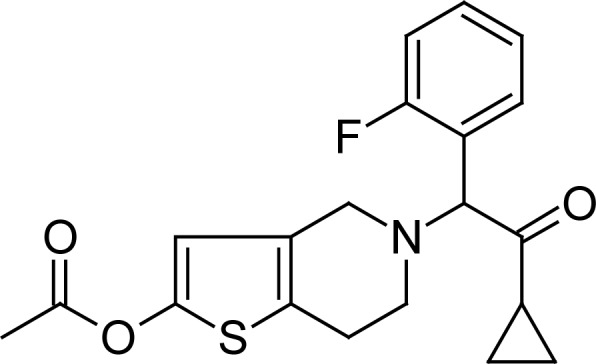
Chemical structure of Prasugrel

Prasugrel (Fig. 1) is chemically (*RS*)-5-[2-cyclopropyl-1-(2-fluorophenyl)-2-oxoethyl]-4,5,6,7-tetrahydrothieno[3,2-*c*]pyridin-2-yl acetate. Prasugrel is a novel third-generation oral thienopyridine that reduces the tendency of platelets, the blood particles responsible for clotting, from sticking or clumping together. Prasugrel blocks a specific receptor on the platelet surface, P2Y12 adenosine diphosphate (ADP), preventing platelets from clumping, which can result in clogged arteries and may lead to a heart attack. Prasugrel is more effective by preventing ischemic events in patients with acute coronary syndrome undergoing percutaneous coronary intervention, increase in bleeding and improved net clinical outcome [1–8].

Forced degradation or stress testing is undertaken to demonstrate specificity for developing stability-indicating methods, particularly when little information is available about potential degradation products. The ICH guideline entitled “Stability Testing of New Drug Substances and Products” requires the stress testing to be carried out to elucidate the inherent stability characteristics of the active substances. The aim of stability testing is to prove how the quality of a drug substance or drug product varies with time under the influence of a variety of environmental factors such as temperature, humidity and light. It enables recommendation of storage conditions, retest period and shelf lives to be established. Regulatory agencies recommend the use of stability-indicating methods for the assay analysis of stability samples. Thus, stress studies are required in order to generate the stressed samples, method development and its validation [9]. Forced degradation of prasugrel was performed under stress conditions (acid, alkaline, photolytic, thermal and oxidative). To establish the stability-indicating nature of the method, stressed samples were analyzed by the proposed method. The proposed RP-HPLC method was validated by assessing its specificity, linearity, accuracy, precision, limits of detection and quantification, system suitability parameters, ruggedness and robustness.

Literature survey reveals that three methods are reported for the assay of prasugrel in human serum by using LCMS [10–12]. Previous investigators [13–16] have also reported the assay of prasugrel in pharmaceutical dosage form by using various techniques such as HPLC and spectrometry. But none of the reported HPLC method is stability-indicating. In the present study attempts were made to develop a rapid, economical, precise and accurate HPLC method for the estimation of prasugrel in the presence of its degradants.

## Experimental

### Chemicals and Reagents

Prasugrel reference standard was provided as a gift sample by Lupin Pharmaceutical Ltd, Mumbai, India. HPLC grade acetonitrile was purchased from Rankem, India. Ammonium acetate, acetic acid and hydrogen peroxide was purchased from Qualigens Fine chemicals, India and sodium hydroxide was purchased from Merck Ltd. India. High pure water was prepared by using Millipore Milli Q plus purification system. 0.45-Pump nylon filter was obtained from Advanced Micro devices Pvt. Ltd. (Ambala Cantt, India). Commercial formulations Prasudoc tablet containing 10mg of prasugrel were purchased from the local market. Other chemicals used were of analytical or HPLC grade.

### HPLC instrumentation and Chromatographic Conditions

The chromatographic system used was Agilent – 1100 series comprised of degasser, quaternary pump, auto injector, column compartment, photodiode array detector and the system was controlled through Chemstation software. Zorbax XDB C_8_ 150 × 4.6 mm, 3.5μm (Agilent Technologies, USA) column maintained at 30°C using column oven, eluted with mobile phase at the flow rate of 1.0 ml/min. The mobile phase consists of aqueous solution of 0.05 M ammonium acetate pH 4.5 with acetic acid -acetonitrile (40:60 *v/v*). The mobile phase filtered through 0.45μm nylon filter and degassed in ultrasonic bath prior to use. Measurements were made with injection volume 10 μL and ultraviolet (UV) detection at 254 nm. For analysis of forced degradation samples, the photodiode array detector was used in scan mode with a scan range of 200–400 nm. The peak homogeneity was expressed in terms of peak purity and was obtained directly from the spectral analysis report using the aforementioned software.

### Standard Preparations

Standard stock solution (1 mg/ml) was prepared by dissolving the drug in the diluents, and standard solution was prepared by diluting them to the desired concentration. Diluent used for preparation was composed of water and acetonitrile in the ratio of 70:30 (*v/v*).

### Preparation of sample Solutions

20 tablets were taken, and their average weight was calculated. The tablets were crushed to a fine powder; drug equivalent to 50 mg powder was transferred to a 50 ml volumetric flask. To this flask 25 ml of diluent was added and the solution was sonicated for 10 min. with intermittent shaking. The volume is makeup with diluent and centrifuged at 10,000 rpm for 10 min. The centrifuged solution was filtered through 0.45μ filter. From the filtered solution, 5 ml of solution was transferred into a 25 ml volumetric flask and diluted to requisite volume with diluent.

### Procedure for forced degradation study

Forced degradation of the drug product was carried out under thermolytic, photolytic, acid, base hydrolytic and oxidative stress conditions.

#### Acidic degradation

Weighed and transferred powder equivalent to 20 mg of prasugrel to 10 ml volumetric flask. Add 3 ml 1N HCl and keep the mixture at 60°C for 3 h in water bath. Allow the solution to attend ambient temperature, then neutralized the solution with 1N NaOH to pH 7 and the volume is make up with diluent. Further dilute this solution to achieve the concentration 200 μg/ml prasugrel.

#### Alkali degradation

Weighed and transferred powder equivalent to 20 mg of prasugrel to 10 ml volumetric flask. Added 3 ml diluent and 2 ml 0.01N NaOH and kept the mixture at room temperature for 30 min. Neutralized the solution with 0.01N HCl to pH 7 and the volume was made up with diluent. This solution was diluted to achieve the concentration 200 μg/ml of prasugrel.

#### Oxidative degradation

Weighed and transferred powder equivalent to 20 mg of prasugrel to 10 ml volumetric flask. Added 3 ml diluent and 2 ml 30% hydrogen peroxide and kept the mixture at 60°C for 1 h in water bath. Allowed the solution to attend ambient temperature and the volume was made up with diluent. This solution was diluted to achieve the concentration 200 μg/ml of prasugrel.

#### Thermal degradation

Weighed powder equivalent to 20 mg of prasugrel and kept in Petri dish at 90°C for 24 h. The solution was prepared to achieve 200 μg/ml of prasugrel.

#### UV-Short (254 nm) degradation

About 200 mg of drug product powder were exposed to UV short light for 24 h. The solution was prepared in diluent to achieve 200 μg/ml of prasugrel.

#### UV-Long (366 nm) degradation

About 200 mg of drug product powder were exposed to UV long light for 24 h. The solution was prepared in diluent to achieve 200 μg/ml of prasugrel.

## Results and Discussion

### Optimization of the chromatographic conditions

To develop a stability-indicating method, it is necessary to separate the analyte peak from degradants peaks. To achieve this, different column stationary phases like C18, CN, C8 tried with different mobile phases containing buffers like phosphate, ammonium acetate and trifluoroacetic acid with different pH (3–9) and organic modifier (acetonitrile) were used.

Our objective of chromatographic method development was to achieve peak tailing factor <2, retention time in between 3 to 10 min. The chromatographic separation of prasugrel from its degradants was achieved using Zorbax XDB C_8_, (150 × 4.6 mm, 3.5μm.) column. In all aforementioned trials, Zorbax XDB C_8_ column shows better performance as compared to other C18 or CN columns. It was determined that aqueous solution of 0.05 M ammonium acetate pH 4.5 with acetic acid and acetonitrile in the ratio of 40:60 (*v/v*), the flow rate of mobile phase at 1.0 ml/min and column temperature at 30°C is the optimal condition. It was observed that pH 4.5 of buffer solution helps to reduce the tailing of analyte peak and to increase the efficiency in terms of theoretical plates. The analyte peak shape with less tailing resolved from degradants and the chromatographic analysis time was found to be less than 10 min. In optimized conditions its degradants were well separated. Typical retention time of prasugrel peak is about 7 min. Method development results are shown in Table 1.

### Results of forced degradation study

Though conditions used for forced degradation were attenuated to achieve degradation in the range of 10–30%, this could not be achieved in case of photolytic degradation even after exposure for prolonged duration [18]. During the initial forced degradation experiments, it was observed that alkali hydrolysis was a fast reaction for almost complete degradation occurred when 0.5N NaOH solution is used. The drug product showed extensive degradation in acid hydrolysis, alkali hydrolysis and oxidative and thermal condition. The hydrolytic degradation observed in both alkali and acidic condition may be due to the presence of carboxyl group into the structure, which results in formation of polar degradants (acid). Also in acidic conditions nitrogen containing ring may undergo protonation and by cleavage it will form polar impurities. Hence, we observed the polar impurities in acid and alkali degradation. Similar type of degradation was reported in clopidogrel which belongs to same class thienopyridine [19, 20]. Also in oxidative condition, reactive sites present in compound oxidized and formed different degradation products. In thermal condition, at moderate temperature no degradation was observed; but, as we employed harsh conditions over a long period, it leads to degradation of prasugrel. Table 2 indicates the extent of degradation, peak purity and assay of prasugrel under various stress conditions. Chromatographic peak purity data was obtained from the spectral analysis report. The peak purity value was found to be greater than 990, indicating a homogeneous peak and thus establishing the specificity of assay method. Fig. 2a to 2f shows the chromatograms of tablet solution, diluent, alkali hydrolysis degradation, oxidative degradation, acid hydrolysis degradation and thermal degradation, respectively. Fig. 3a to 3d shows peak purity spectra of prasugrel in presence of it degradants. Assay of prasugrel was unaffected by the presence of other degradants which confirms the stability-indicating power of the method.

## Method validation

### Specificity

Specificity is the ability to measure accurately and specifically the analyte of interest in the presence of other components that may be expected to be present in the sample matrix. It was found that the proposed method was specific because there is no interference of diluent and excipients ensuring that the peak response is due to only a single component. Photodiode array detection was used as an evidence of the specificity of the method and to evaluate the homogeneity of the analyte peak. The peak purity values that are more than 999 for drug product show that the peaks of analyte were pure. Formulation excipients and degradants were also not interfering with the analyte peak.

### Calibration and linearity:

Linearity experiment was tested from range of 10 to 200% of the targeted level assay concentration 200 μg/ml. The linearity solutions were injected in triplicate. The calibration graph was obtained by plotting peak area against the concentration of the drug. The equation of the calibration curve y = 9.327x + 0.7701. The calibration graph was found to be linear in the aforementioned concentrations with correlation coefficient 0.999.

### Precision (repeatability):

The precision of the proposed method was evaluated by carrying out six independent (200μg/ml) assays of test sample. RSD (%) of six assay values was calculated. Intermediate precision was carried out by analyzing the samples by different analyst on another instrument. Table 3 indicates the results of the precision study, which shows the method is reliable (RSD %< 2).

#### Accuracy (recovery test)

Accuracy of the method was studied by recovery experiments. The recovery experiments were performed by adding known amounts of drug in the placebo. The recovery was performed at three levels: 80%, 100% and 120% of the label claim of the tablet (10 mg of prasugrel). The recovery samples were prepared as per the procedure mentioned in preparation of sample. Three samples were prepared for each recovery level. The solutions were then analyzed, and the percentage recoveries were calculated. The recovery value for prasugrel ranged from 98.88 to 100.59%. The average recovery of three levels (nine determinations) was 99.76%. Results are shown in Table 4.

### Robustness

The robustness is the ability of method to remain unaffected by small changes in parameters. To determine robustness of the method, experimental conditions were purposely altered and assay, peak tailing, theoretical plates and peak area %RSD were evaluated. The flow rate of the mobile phase was 1.0 ml/min. To study the effect of flow rate it was changed to 0.1 units from 1.0 to 0.9 ml/min and 1.1 ml/min. The effect of column temperature was studied at 28°C and 32°C instead of 30°C, while other mobile phase components were kept constant. The effect of mobile phase composition was studied in aqueous solution of 0.05 M ammonium acetate pH 4.5 with acetic acid: acetonitrile (38:62 *v/v*) and (42:58 *v/v*). At all conditions sample was assayed in triplicate. The effect of detection wavelength was studied at 250 nm and 258 nm. Assay % at all deliberate conditions within 98.81 to 99.88 %. Results are shown in table 5.

### Determination of limit of quantification and limit of detection (LOQ & LOD)

The detection and quantification limits were evaluated from calibration curves plotted in concentration range of detection level–300μg/ml. The formula used were LOD= 3.3σ/S and LOQ= 10σ/S (where σ = standard deviation of response and S = slope of calibration curve). LOD and LOQ for this method were found to be 0.110 and 0.364, respectively. These values indicated the method was very sensitive to quantify the drug.

The standard drug solutions for each value of LOD and LOQ concentration were injected 6 times. % RSD values for the area of replicate injections were calculated.

### Solution stability

The stability of standard solution was tested at the intervals of 12 and 24 h. The stability of solutions was determined by comparing results of area% and peak purity of prasugrel. The area% values were found to be within 0.5 % after 24 h. The results indicate that the solution was stable for 24 h at ambient temperature as there was no formation of any unknown peak and solution remains stable. The RSD of peak area% and peak purity were 0.32%, 999.983, respectively.

## Conclusion

The LC method described here is a very simple, sensitive and accurate procedure for the estimation of prasugrel. The developed and validated LC method is stability-indicating and enables specific, accurate, robust and precise analysis of prasugrel in formulation. The method is sensitive enough for quantitative detection of the analyte in pharmaceutical preparations. Thus the proposed method can be used for routine analysis in quality control departments and for studies of the stability of pharmaceutical tablets. The validation data indicate good precision, accuracy and reliability of the method. The sample recoveries in formulations were in agreement with their respective label claim and suggested noninterference of formulation excipients in the estimation of prasugrel.

## Figures and Tables

**Fig. 2a.**
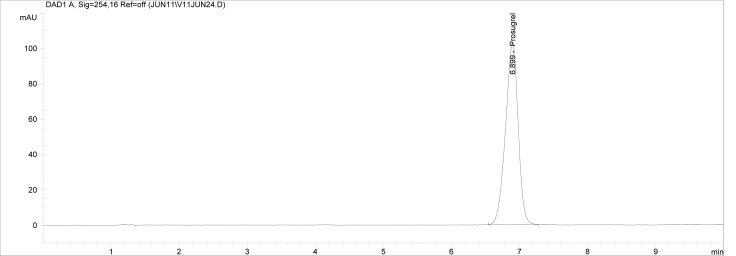
Chromatogram of Prasugrel tablet solution.

**Fig. 2b.**
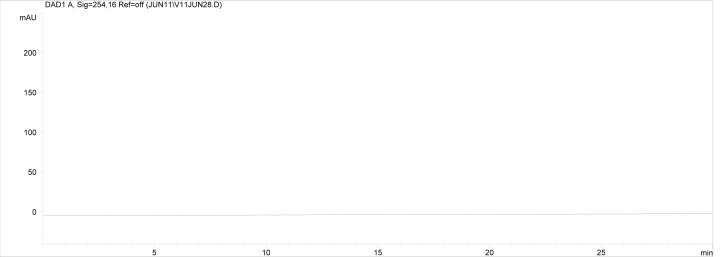
Chromatogram of blank solution.

**Fig. 2c.**
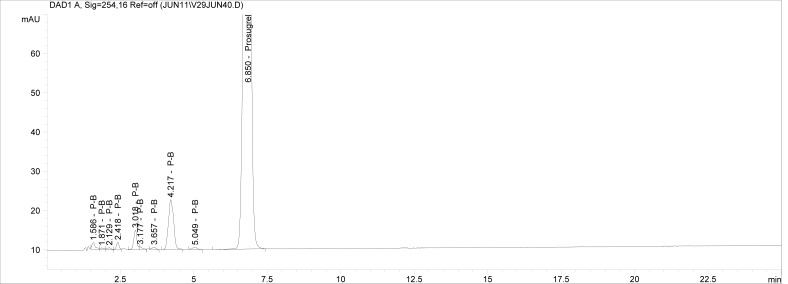
Chromatogram of prasugrel alkali hydrolysis degradation.

**Fig. 2d.**
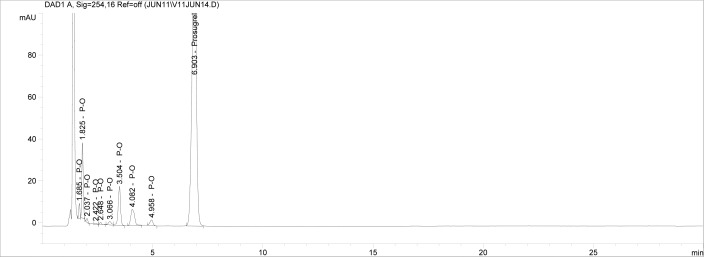
Chromatogram of oxidative degradation.

**Fig. 2e.**
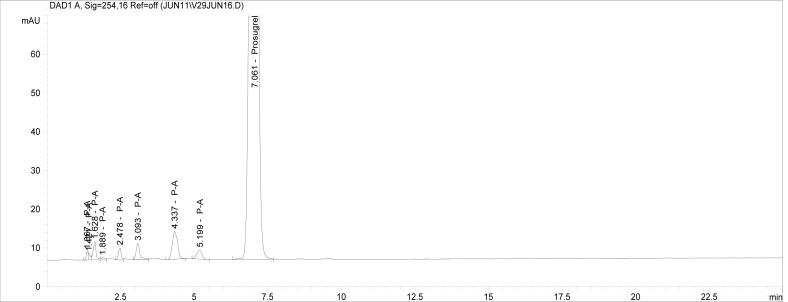
Chromatogram of acid hydrolysis degradation.

**Fig. 2f.**
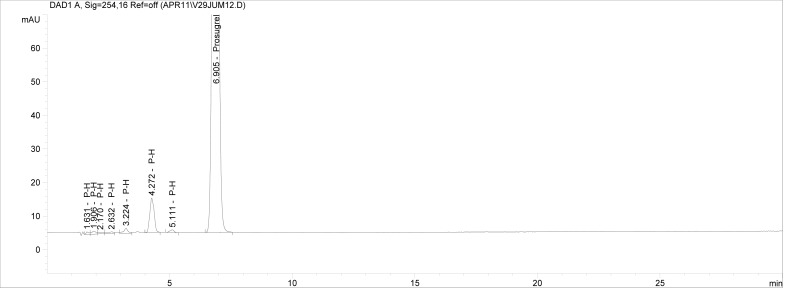
Chromatogram of thermal degradation.

**Fig. 3a.**
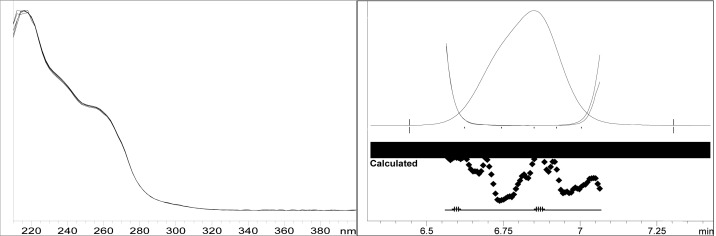
Peak purity spectra of prasugrel in alkali hydrolysis degradation

**Fig. 3b.**
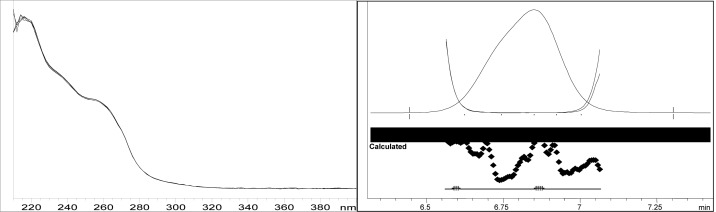
Peak purity spectra of prasugrel in oxidative degradation

**Fig. 3c.**
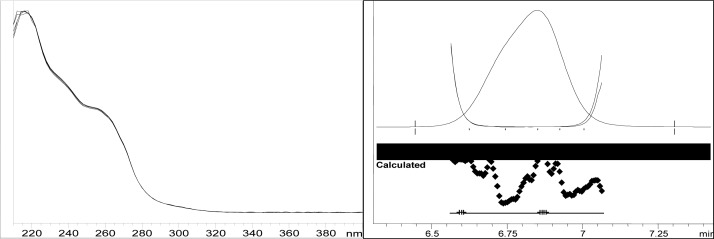
Peak purity spectra of prasugrel in acid hydrolysis degradation

**Fig. 3d.**
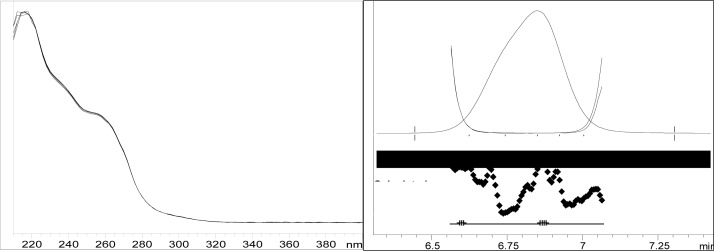
Peak purity spectra of prasugrel in thermal degradation

**Tab. 1.** Results of method development.

**Column**	**Retention time (min)**	**Theoretical Plates**	**USP Tailing**
YMC ODS A C18;	13.2	4723	1.6
25cm × 4.6 mm *d.,*5 μ			
Altims CN;	5.2	3523	1.3
25cm × 4.6 mm *d.,*5 μ			
Zorbax XDB C8;	6.0	7023	1.1
15cm × 4.6 mm *d.,*3.5 μ			

**Tab. 2.** Results of analysis of forced degradation study

**Stress condition**	**Degradation (%)**	**Peak purity^a^**	**Assay (%)**
Acidic (60°C/3 hr)	11.33	999.915	88.38
Alkali (RT/30 min)	13.38	999.743	86.26
Oxidative (60°C/1hr)	19.18	999.592	81.23
Thermal (90°C/24hr)	6.11	999.831	93.13
UV-short (24 hr)	No degradation	999.901	99.33
UV-long (24hr)	No degradation	999.925	99.69

a peak purity values in the range of 990–1000 indicate a homogeneous peak.

**Tab. 3.** Precision data

**Analyst**	**Assay (%)** **(n=6)**	**% RSD**
Analyst-1	99.23	0.59
Analyst-2	99.63	0.86

**Tab. 4.** Results of Accuracy experiment using proposed method.

**Level (%)**	**Amount of drug spiked (mg)**	**Found**	**Recovery%**

		**(mg)**	**(n=3)**
80	8.13	8.09	99.39
100	10.19	10.2	100.12
120	12.35	12.33	99.78

**Tab. 5.** Results of robustness study.

**Sr. no.**	**Parameter**	**Variation**	**Assay % (n=3)**
1.	Flow rate (± 10% of the set flow)	a) At 0.9 ml/min b) At 1.1 ml/min	99.56 99.18
2.	Mobile phase composition (± 2% of organic modifier)	a) At 62 ml b) At 58 ml	98.97 99.32
3.	Temperature (± 2° C of set temperature)	a) At 28°C b) At 32°C	98.81 99.23
4.	Wavelength (± 4 nm)	a) At 250 nm b) At 258 nm	99.88 99.75
